# Report of the draft genome sequences of human sapovirus from children with acute gastroenteritis in Malawi, Southern Africa

**DOI:** 10.1128/mra.00966-25

**Published:** 2025-11-17

**Authors:** Flywell Kawonga, Ernest Matambo, End Chinyama, Chimwemwe Mhango, Clara Majengo, Josephine Msowoya, Prisca Benedicto-Matambo, Benjamin Kumwenda, Celeste M. Donato, Arox W. Kamng'ona, Milton T. Mogotsi, Nkosazana Shange, Ayodeji E. Ogunbayo, Francis E. Dennis, Martin M. Nyaga, Chrispin Chaguza, Khuzwayo C. Jere

**Affiliations:** 1 Malawi-Liverpool-Wellcome Programme, Queen Elizabeth Central Hospital 560808, Blantyre, Malawi; 2 Faculty of Health and Life Sciences, Institute of Infection, Veterinary & Ecological Sciences, University of Liverpool 4591, Liverpool, United Kingdom; 3 Department of Medical Laboratory Sciences, Faculty of Biomedical Sciences and Health Profession, Kamuzu University of Health Sciences 37610, Blantyre, Malawi; 4 Department of Pharmacy, School of Life Sciences and Allied Health Professions, Kamuzu University of Health Sciences 37610, Blantyre, Malawi; 5 Department of Pathology, School of Medicine and Oral Health, Kamuzu University of Health Sciences 37610, Blantyre, Malawi; 6 Biomedical Sciences Department, School of Life Sciences and Allied Health Professions, Kamuzu University of Health Sciences 37610, Blantyre, Malawi; 7 Enteric Diseases Group, Murdoch Children's Research Institute 34361, Parkville, Australia; 8 Next Generation Sequencing Unit, Faculty of Health Sciences, School of Biomedical Sciences and Division of Virology, University of the Free State, Bloemfontein, South Africa; 9 Department of Electron Microscopy and Histopathology, Noguchi Memorial Institute for Medical Research, University of Ghana 58835, Accra, Ghana; 10 Department of Host-Microbe Interactions, St Jude Children's Research Hospital, Memphis, Tennessee, USA; Queens College, Queens, New York, USA

**Keywords:** human sapovirus (SaV), acute gastroenteritis, genome sequencing, Malawi, SACEV, Caliciviridae, enteric viruses

## Abstract

Human sapoviruses are increasingly recognized as a cause of acute gastroenteritis in children worldwide but remain poorly studied, particularly in African settings. Here, we report the four draft genome sequences of human sapovirus from Malawi, Southern Africa, collected from children with acute gastroenteritis.

## ANNOUNCEMENT

Human sapovirus (SaV), a member of the *Caliciviridae* family, is a significant cause of acute pediatric viral gastroenteritis, particularly in low- and middle-income countries (LMICs). There are at least 19 SaV genogroups, four of which infect humans (genogroups GI, GII, GIV, and GV) (1). SaVs have a single-stranded, positive sense ribonucleic acid (RNA) genome of approximately 7.5 kb, typically comprising two or three open reading frames (ORFs), namely ORF1, ORF2, and sometimes ORF3 (2, 3). SaVs are recognized as a major cause of childhood diarrhea (4, 5) but remain genomically under-characterized in Africa. Malawi, a low-income country with a high SaV burden (6), still lacks sufficient sequence data. This study presents four draft genome sequences of SaV isolated from children under 5 years of age with acute gastroenteritis at two hospitals in Blantyre, Malawi, between 2012 and 2024, as part of a project aimed at sequencing and characterizing the antigenic diversity of enteric viruses in Africa.

Selected stool samples collected each month were screened for SaV and other enteric viruses using customized real-time PCR–based TaqMan Array Cards (TACs), as previously described (7). Total RNA was extracted from SaV-positive samples with PCR threshold (Ct) <35 using the QIAamp Viral RNA Mini Kit (Qiagen, Germany). Complementary DNA (cDNA) was synthesized using QIAseq FX Single Cell RNA Library Kit (Qiagen, Germany). The SaV genomes described here were generated by shotgun metagenomic sequencing, which produces genomic sequences of all the genetic material present in the sample. Genomic libraries were prepared using the Illumina DNA Prep kit (Illumina, USA) before being sequenced on Illumina NextSeq 2000 platform using a P1 flow cell and 300-cycle reagent kit (2 × 150 bp paired-end reads). Quality control of the sequenced genomes was performed using FastQC v0.11.7 (https://www.bioinformatics.babraham.ac.uk/projects/fastqc/).

**Fig 1:**
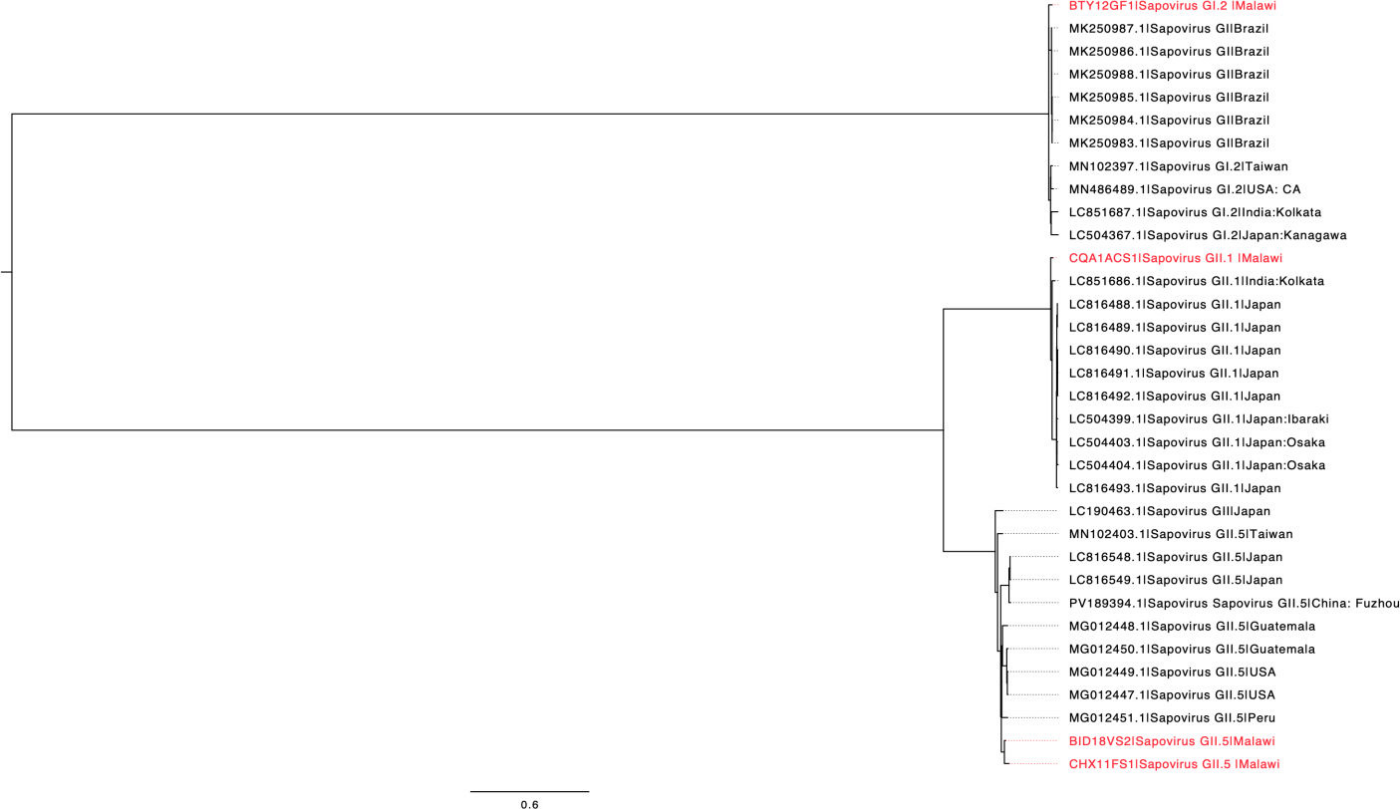
Contextual genomes of human SaV were retrieved from the NCBI virus database, selected based on complete coding sequences and broad global representation. Multiple sequence alignment was performed using MAFFT v7 with default parameters, and a maximum likelihood phylogenetic tree was inferred using IQ-TREE V2.4.0 with the GTR + G substitution model. Branch support was assessed using 1,000 ultrafast bootstrap replicates. The resulting tree was annotated in FigTree v1.4.4 and mid-point rooted to establish evolutionary directionality. Metadata associated with each strain was used to annotate the tree tips, with Malawian strains highlighted in red and contextual genomes from NCBI shown in black.

Low-quality reads were trimmed using Trimmomatic (v0.39) (8). To eliminate contaminating human reads, the trimmed sequences were aligned against the human genome (Homo_sapiens.GRCh38.dna.primary_assembly.fa, NCBI accession ID: GCA_000001405.15) using Bowtie2 v2.5.4 (9). The non-human reads were mapped to the reference genomes listed in Table 1 using the Burrows–Wheeler Aligner (BWA, version 0.7.18-88 r1243-dirty) (10). Using iVar (version 1.4.4), high-confidence bases were called from the aligned sequence reads to produce the consensus genomes (11). Genome assemblies were subsequently annotated using Prokka (version 1.14.6) (12). BLASTn analysis showed that CQA1ACS1, CHX11FS1, BTY12GF1, and BID18VS2 shared 96.70%, 93.91%, 96.75%, and 95.04% nucleotide identity with reference genomes as depicted in Table 1, respectively. Genome assembly depth was assessed using Samtools (v1.21). (https://www.htslib.org/). Table 1 summarizes the read and assembly characteristics, highlighting key genomic features for all sequenced samples. Genome assembly identity was confirmed as SaV using Genome Detective (https://www.genomedetective.com/app/typingtool/virus/). Genotyping was performed using the Human Calicivirus Typing (HuCaT) tool (13), which classified the Malawian SaV genomes into genogroups GII.1, GII.5, GI.2, and GII.5. Phylogenetic analysis revealed that two Malawian GII.5 strains clustered together, while GI.2 and GII.1 strains clustered with genomes from Brazil and India, respectively (Fig. 1). All analyses were carried out using default settings unless otherwise specified.

**TABLE 1:** Overview of assembly results for all sequenced samples, highlighting key genomic features

Sample ID	CQA1ACS1	CHX11FS1	BTY12GF1	BID18VS2
Date of collection	20/03/2024	15/06/2019	23/02/2015	01/02/2013
Sample type	Stool	Stool	Stool	Stool
Cycle threshold (Ct)	28	26	23	28
Sequencing platform	NextSeq 2000	NextSeq 2000	NextSeq 2000	NextSeq 2000
Reads	Paired	Paired	Paired	Paired
Raw sequence length (bp)	36-151	36-151	36-151	36-151
Total number of reads generated	785899	336488	480965	603603
Total mapped reads to the reference	26292	10747	88690	45113
Sequencing depth	384.335 X	154.502 X	1285.79 X	644.01 X
Reference Genome for Read Mapping	MG012405.1	MG012448.1	LC816518.1	MG012448.1
Genome length (bp)	7417	7405	7474	7385
GC content (%)	52.1	51.6	48.6	51.6
Reference identity NT	96.70%	93.91%	96.75%	95.04%
BLASTn Top Hit (NCBI Accession)	LC851686.1	MG012448.1	MK250987.1	MG012448.1
Genotype	GII.1	GII.5	GI.2	GII.5
NCBI Accession	PV872135	PV872134	PV872133	PV872132
Sequence Read Archive	SRA-CQA1ACS1	SRA-CHX11FS1	SRA-BTY12GF1	SRA-BID18VS2

## Data Availability

The SaV genomes from this study have been deposited in GenBank under accession numbers PV872132, PV872133, PV872134, and PV872135. The Sequence Read Archive is available under BioProject Accession PRJNA1276427.
